# The Building Wealth and Health Network: methods and baseline characteristics from a randomized controlled trial for families with young children participating in temporary assistance for needy families (TANF)

**DOI:** 10.1186/s12889-016-3233-4

**Published:** 2016-07-16

**Authors:** Jing Sun, Falguni Patel, Rachel Kirzner, Nijah Newton-Famous, Constance Owens, Seth L. Welles, Mariana Chilton

**Affiliations:** Department of Health Management and Policy, Drexel University Dornsife School of Public Health, 3600 Market Street, 7th Floor, Philadelphia, PA 19104 USA; Department of Epidemiology and Biostatistics, Drexel University Dornsife School of Public Health, 3215 Market Street, 5th Floor, Room 535, Philadelphia, PA 19104 USA; Department of Epidemiology, Johns Hopkins University Bloomberg School of Public Health, 2213 McElderry St, Room M139, Baltimore, MD 21205 USA; Stockton University, Galloway, NJ 08205-9441 USA

**Keywords:** Food insecurity, Adverse childhood experiences, Violence, Trauma, Poverty, TANF, Assets, Depression, Randomized controlled trial

## Abstract

**Background:**

Families with children under age six participating in the Temporary Assistance for Needy Families Program (TANF) must participate in work-related activities for 20 h per week. However, due to financial hardship, poor health, and exposure to violence and adversity, families may experience great difficulty in reaching self-sufficiency. The purpose of this report is to describe study design and baseline findings of a trauma-informed financial empowerment and peer support intervention meant to mitigate these hardships.

**Methods:**

We conducted a randomized controlled trial of a 28-week intervention called Building Wealth and Health Network to improve financial security and maternal and child health among caregivers participating in TANF. Participants, recruited from County Assistance offices in Philadelphia, PA, were randomized into two intervention groups (partial and full) and one control group. Participants completed questionnaires at baseline to assess career readiness, economic hardship, health and wellbeing, exposure to adversity and violence, and interaction with criminal justice systems.

**Results:**

Baseline characteristics demonstrate that among 103 participants, there were no significant differences by group. Mean age of participants was 25 years, and youngest child was 30 months. The majority of participants were women (94.2 %), never married (83.5 %), unemployed (94.2 %), and without a bank account (66.0 %). Many reported economic hardship (32.0 % very low household food secure, 65.0 % housing insecure, and 31.1 % severe energy insecure), and depression (57.3 %). Exposure to adversity was prevalent, where 38.8 % reported four or more Adverse Childhood Experiences including abuse, neglect and household dysfunction. In terms of community violence, 64.7 % saw a seriously wounded person after an incident of violence, and 27.2 % had seen someone killed. Finally, 14.6 % spent time in an adult correctional institution, and 48.5 % of the fathers of the youngest child spent time in prison.

**Conclusions:**

Baseline findings demonstrate that caregivers participating in TANF have suffered significant childhood adversity, adult violence exposure, and poverty-related stressors that can limit workforce success. High prevalence of housing and food insecurity, exposure to adversity, violence and criminal justice systems demands comprehensive programming to support families. Trauma-informed approaches to career readiness such as the Building Wealth and Health Network offer opportunities for potential success in the workforce.

**Trial registration:**

This study is retrospectively registered with ClinicalTrials.gov

The Identifier is: NCT02577705

The Registration date is October 13, 2015

## Background

Families with young children under age six participating in the Temporary Assistance for Needy Families Program (TANF) that are deemed “work mandatory” are required to participate in work-related activities for at least 20 h per week in order to receive TANF benefits. However, due to financial hardship, poor health, and exposure to violence and adversity, the success families achieve through TANF participation can be limited. In partnership with the state of Pennsylvania Department of Human Services, this randomized control trial pilot, The Building Wealth and Health Network (The Network RCT), is an ongoing study that seeks to evaluate effectiveness of an innovative intervention to address family hardships associated with exposure to adversity and violence, social isolation, and low financial capability in order to help families get on the pathway to self-sufficiency. This program is also meant to test a workforce development model that includes attention to mental, emotional, and financial health that can become a new model for TANF education and training programming. The Network RCT is a 28-week educational program with a full intervention group, a partial intervention group, and a control group. The full intervention includes helping participants to open a savings account, into which participants are provided a 1:1 match of up to 20 dollars a month; financial empowerment education; and trauma-informed peer support. This counts toward 6 h of work participation per week. The partial intervention includes the 1:1 matched savings accounts and financial education, counting for three hours of work participation per week. Network RCT staff document these hours along with other work participation hours up to 20 h per week as reported by participants, in the state data management system, Commonwealth Workforce Development System (CWDS). The control group participates in TANF activities as prescribed by County Assistance Income Maintenance Workers. This primarily consists of being required to participate in 20 h per week of state-supervised TANF mandated work participation or supervised job search activities.

Below, we describe The Network RCT research methods and program design. We also describe baseline characteristics of the sample of 103 participants, all of whom have a young child under age six and who were deemed to be required to comply with the 20 h per week work participation requirement. We describe their career readiness, hardship (food insecurity, housing insecurity and energy insecurity), self-rated health and depression, and exposure to violence and adversity, including history of incarceration. Baseline measures demonstrate that TANF participants with young children who are originally deemed “work mandatory” report a complex picture of career readiness. Additionally, they report high rates of financial hardship in relation to food, housing, and utilities, high rates of depression and poor health, developmental risk among their children, significant exposure to community violence, high rates of adverse childhood experiences, and a significant history of incarceration. Research has shown that TANF programs that prioritize health and wellbeing have a positive influence on helping families to find and keep employment and demonstrate movement towards self-sufficiency [1–5]. Rarely, however, are characteristics related to deep hardship such as homelessness and hunger coupled with high rates of violence exposure and incarceration taken into account in TANF education and training programs, nor do federal guidelines call for improving such metrics beyond employment and exit from TANF. We frame these hardship experiences as indicators of exposure to trauma, and suggest that TANF programs be built to explicitly address these hardships and integrate trauma-informed peer support approaches into job training and skill building. A trauma-informed intervention such as the Building Wealth and Health Network can not only improve mental health and wellbeing and create a path to self-sufficiency, but also, we suggest that given the theory developed around helping individuals overcome trauma and related isolation, peer support is a key component to any type of programming meant to have a positive impact on the health and wellbeing of caregivers’ young children [6–9].

In 2013, 45.3 million people lived in poverty in the United States, including over one in five children under the age of six, yet only 27 % of eligible families received TANF. In Pennsylvania, the number of people living in poverty is slightly higher than the national average but still only 31 % of those eligible, received TANF in 2013 [10]. While child poverty increases the risk of poor health and developmental delays [11], many public assistance programs, such as the Supplemental Nutrition Assistance Program (SNAP), the Special Supplemental Nutrition Program for Women, Infants, and Children (WIC), and housing subsidies, protect vulnerable children from the negative effects of poverty [12, 13]. However, it is unclear if TANF has demonstrated significant improvements in maternal and child wellbeing, in moving families out of poverty, or in fully preparing low-income families for success in the workforce [14, 15].

One of the goals of TANF is to provide job skills and education programs to support adults and their children as they enter the workforce. However, many families experience barriers to employment, which may prevent them from successfully transitioning off of TANF. This may be due in part to poor health among those receiving TANF, as approximately one third of TANF recipients have reported a work-limiting health condition [4, 5, 16]; and almost 43 % of TANF recipients reported multiple types of disability including memory impairment, emotional/mental limitations, movement limitations, and sensory impairment [2]. In addition to the poor health and disability reported by TANF participants, they also report alarmingly high rates of exposure to violence and adversity in their communities and in their family relationships [17–20]. For instance, among TANF eligible families, rates of intimate partner violence are as high as 74 % compared to up to 31 % in the general community [21], posing a major barrier to employment [22–25]. While exposure to violence in adulthood indicates severe hardship, such exposure to violence across the lifespan, reaching back into childhood, is also reported at significantly higher rates among low-income families. Adverse childhood experiences (ACEs) consisting of physical and emotional abuse and neglect, sexual abuse, and household dysfunction, such as having a household member in prison, or witnessing domestic violence are especially prevalent among those receiving TANF [26]. ACEs and violence exposure are closely linked to work-limiting conditions such as depression, cardiovascular disease, food insecurity and other health conditions [27–30]. Exposure to ACEs has been linked to higher rates of worker absenteeism and stress surrounding work and finances in adulthood, indicating an association between ACEs and later financial stability [31]. Other related barriers to work are having a criminal record, or having served time in prison [16], and among female heads of household TANF recipients, the prevalence of interactions with the criminal justice system is quite high compared to other low income populations [16, 32]. Finally, when a parent of a young child is in prison, it can have detrimental effects on the child’s development, which in turn, demands more attention, time and care by adult caregivers, creating more barriers to work [33]. Female heads of households who have a criminal history are at greater risk for reaching the federal time limit of 60 months of TANF receipt, which can exacerbate the barriers of obtaining employment [34]. Furthermore, children whose parents have a criminal history are at a greater risk of becoming involved in the criminal justice system and are more likely to exhibit high-risk behaviors than children in the general population [32].

High levels of adversity among TANF recipients and those living in poverty is a significant concern because adversity impacts physical and mental health [29, 35], academic achievement [36], employment [37], the development of executive skills such as working memory and cognitive controls [38, 39], and parenting of the next generation [40]. Emphasis on job search and work participation for families without attention to poor health and adversity can be a set up for failure. A recent RCT in Florida found that women with chronic health conditions receiving TANF, while deemed mandatory to work had greater difficulty attaining employment than women with the same characteristics who received Public Health Nursing care related to their health condition [4]. In addition, other investigators have found that social support and comprehensive approaches to social work that build resilience may have success in limiting the negative impacts of exposure to violence and adversity [41, 42]. However, the majority of TANF programs across the country rarely integrate such approaches, and in many states, TANF participants that are unable to meet the mandated work requirements, potentially due to poor health and exposure to violence and adversity, may be more likely to be “sanctioned,” meaning they would either have their cash benefits reduced or cut off completely for a duration of time.

Families that receive sanctions are more likely to have significant health barriers to work participation [43]. Additionally, those who have been sanctioned reported higher rates of intimate partner violence [44], and physical and behavioral health problems [45, 46]. Sanctions can then increase hardships families already face. For instance, compared to families who have not been sanctioned, families that experience sanction report higher rates of household food insecurity [47], utility shut-offs [48], child hospitalizations [47], difficulty paying for health care [49], homelessness [50], and disruptions in children’s schooling [51]. This is especially problematic as those who are sanctioned are more likely to have young children, putting those children at increased risk during sensitive developmental phases [11].

To compound the mental and physical barriers to work and self-sufficiency, TANF-eligible families, like many low-income families, have low financial literacy, poor or no credit history, few or no assets, and are unbanked (having no checking or savings account) or under-banked (having a bank account, but still primarily relying on alternative financial services such as check cashing and money orders) [52–55]. In order to supplement meager income, families may resort to earning income and spending money through the informal economy where they are paying higher fees for check cashing, paying bills, and acquiring loans [56]. This lack of access to mainstream financial institutions and activities can be crippling, as savings and other tangible assets play a critical role in helping shield families from unexpected income shocks, allowing families to weather periods of economic uncertainty without falling further into poverty [57–59]. Savings create a financial foundation, increase economic security, and can, over time, be invested into education for children. Asset building activities show improvements in health, greater civic and community involvement, and lower rates in the intergenerational transfer of poverty [59]. Savings can also reduce the extreme stress that often accompanies maternal depression [60]. Finally, the positive impacts of savings have held true even during the recession [61, 62]. It is true, however, that people who are participating in TANF have a very difficult time opening and maintaining a bank account, or savings account at all. In order to develop a new habit of saving, and to begin to create a seed kernel on which to build assets, even small increments of savings have been identified with ability to envision a future, set goals, and build knowledge and skills, as well as to be associated with positive education outcomes for children [57, 58, 63–66].

Overall, the evidence on effectiveness of education and training programs across the country points to the urgent need to pivot the approaches for education and training by integrating programming that helps to provide social support, build resilience, address health barriers, and overcome exposure to trauma and adversity. Evidence of the need to address trauma among families has become so apparent that the US agency that administers TANF, the Agency for Children and Families, is calling for trauma-informed approaches that incorporate approaches that address two generations (caregiver and child), rather than focusing solely on either caregiver or child [67].

## Methods

### The Building Wealth and Health Network Randomized Controlled Trial (Network RCT)

The Building Wealth and Health Network Randomized Controlled Trial (Network RCT) is built to evaluate a pilot intervention that develops a new model of public benefits provision, meant to leverage participants’ own strengths to become financially self-sufficient. The Network RCT provides asset-building activities and trauma-informed peer support to low-income caregivers of young children under the age of six who are participating in TANF. The goal of the program is to increase the caregiver’s financial, human, and social capital in order to improve financial security and maternal and child health.

### Network RCT research design

#### Population

This study has been approved by the Drexel University Institutional Review Board. Study recruitment took place in June and July of 2014 at three County Assistant Offices (CAOs) in Philadelphia where TANF participants who are residents of South or Southwest Philadelphia come to enroll and/or recertify for benefits. Participants were included in the study if they were at least 18 years old, had been receiving TANF cash assistance for 4 years or less, had at least one child under six years old, and were considered “mandatory to work” for 20 h per week by the federal welfare guidelines. Mandatory means that participants have no documented physical or mental health barriers or documented caregiving responsibilities (for a newborn or a disabled child or adult) that will prevent them from working. Participants were excluded from the study if they had been involved with bank fraud in the past or had a household member already enrolled in the program.

**Fig. 1:**
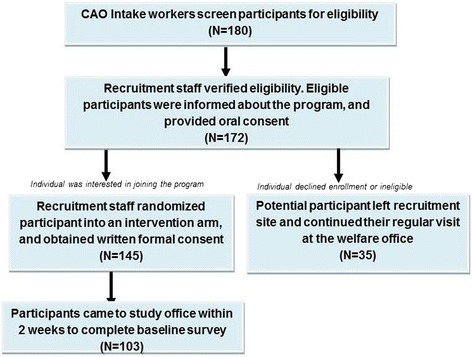
Recruitment and Randomization Procedure

Figure 1 presents the study recruitment procedure. During their visit, TANF participants were screened by CAO Intake Workers for their eligibility. At that time, recruitment staff reconfirmed participants’ eligibility based on TANF participation history and child’s age. No medical or social screening procedures were used. After they verified eligibility, the Intake workers, informed participants about the Building Wealth and Health Network. A total of 180 participants that were present at the CAO during recruitment days were referred to the Network RCT by CAO staff upon initial screening for eligibility. Upon re-screening by study team members, 8 people were determined to be ineligible. Upon explaining the program, 27 people declined to participate and wished to be referred back to the intake worker so that they could enroll in a traditional job-search program, or apply their existing education or employment hours towards the work participation requirement. One hundred forty-five people agreed to participate in the study. All were randomized into the three intervention groups and provided informed consent. After randomization, participants were scheduled to complete the baseline survey at the study site within 14 days. A total of 103 participants arrived for the orientation activities and to complete the baseline survey. This 71 % attendance rate between referral and start of a program is significantly higher than the Pennsylvania state average of 50 % attendance from referral to participation activities [68].

#### Randomization procedures

After participants provided verbal informed consent, recruitment staff assigned a study ID to participants. Study IDs were 150 random numbers ranging from 1 to 10,000 which were previously assigned to the three study groups, so that each participant was randomly assigned to a particular study group. After randomization, the recruitment staff then conducted a written informed consent procedure for participants, according to the intervention group to which the participants had been assigned.

We used single blinding among three study groups, and program instructors and facilitators were instructed not to discuss any information about other groups with participants.

#### Data collection procedures

We used the Audio Computer-Assisted Self-Interview (ACASI) method for baseline and follow-up data collection. The ACASI methodology has been widely used both in randomized controlled trials and in surveys that collect information about sensitive topics and behaviors [69, 70]. The ACASI interviews were designed and built through the Questionnaire Development System^TM^ software, which is developed and sold by NOVA Research Company. Participants from each study group take a 1 h survey every 3 months, from baseline until 15 months post-recruitment. Incentives of $25 in cash and lunch or snacks are provided each time. Resources were made available to all participants after the baseline survey, and the option of following up with a social worker is also made available.

### Network RCT program design

#### Full Intervention

Program activities include helping participants to open savings accounts at a local non-profit federal credit union (with 1:1 matches of up to 20 dollars per month) over the course of 12 months; financial empowerment classes using a curriculum developed especially for this program (3 h classes once per week); and peer support groups called Self Empowerment Groups (SEG) that draw from the Sanctuary® trauma-informed approach to social services (two and a half hour sessions once per week) [71]. The SEG curriculum draws key components from the S.E.L.F. tool within Sanctuary, focusing on the four domains: creating personal, emotional, moral and physical safety (S), processing and managing emotions (E), recognizing loss and letting go (L), and developing goals for a sense of future (F). S.E.L.F. establishes a common language that all people who have experienced adversity can use to organize their lives and work towards building stable foundations to support their goals and invest in their potential. To address personal mental health concerns and other health barriers such as housing, food, and safety, the staff Social Worker follows up individually and makes a referral to an appropriate agency that could assist them in meeting their needs.

The Financial Empowerment curriculum consists of interactive exercises, worksheets, and journal assignments to foster understanding and practice of banking, building credit and debt management, making the most of one’s money, and setting financial goals for oneself and one’s family. Content focuses on identifying and harnessing the internal and external resources that participants can leverage to begin taking steps towards financial self-sufficiency. Program group activities are provided for 28 weeks.

Participants are encouraged to make weekly deposits in their account as little or as much as they feel they can for the duration of 12 months. During class sessions, deposits are made in class, with a bank representative available to assist, as well as at branches and ATMs. Understanding that participants relying on TANF income have limited funds to deposit, the goal for the small savings during the program is to launch a practice of asset-building that would carry beyond their participation in TANF. The maximum match of $240 minimizes the possibility that participants exceed the TANF asset-limit of $1000.

#### Partial intervention

This group receives matched savings accounts and financial empowerment education as described above, but does not receive the trauma-informed peer support.

#### Control group

The Control group does not receive assistance in opening a matched savings account, and are required by the CAO to participate in other TANF mandated work participation activities according to standard procedure.

Our hypotheses are that over the course of 15 months, those receiving the full intervention would have greater improvements in depression, self-rated health, savings patterns and financial security as compared to those who receive the partial intervention and the control group; and that those receiving the partial intervention may show improvements in financial security as compared to the control group.

### Primary outcomes

All measurements used in The Network RCT survey, including baseline measures reported here, are listed in Table 1. At baseline, we collected information on demographics and socio-economic status, career readiness, family economic hardship, financial behavior, physical and mental health of the participant and his or her youngest child, exposure to adversity and violence, and incarceration. The social and demographic information included participants’ age, gender, immigration status, residential zip code, race/ethnicity, marital status, sexual orientation, educational attainment, employment status, and banking participation.

**Table 1:** Surveys and Measurements Used in the Building Wealth and Health Network Randomized Controlled Trial Survey

Measurements	Scales	References	Frequency
Demographics and socio-economic status			
Demographics	Gender, age, residential zip code, immigration status, race and ethnicity, marital status, sexual orientation, educational levels	N/A	Baseline only
Current employment	Self-report whether currently working	N/A	Baseline/follow-up
Career Readiness			
Learning Needs^a^	Washington State Learning Needs Screening Tool 13-item survey	Washington State Division of Employment and Social Services, 1997 [111]	Baseline only
Employment Hope	Derived from the Employment Hope Scale 9-item survey	Hong, et al. 2013 [72, 73]	Baseline/follow-up
Self-Efficacy	General Self-Efficacy Scale 10-item survey	Schwarzer, et al. 1995 [74]	Baseline/follow-up
Family economic hardship			
Food Insecurity	U.S. Household Food Security Survey Module	Bickel, et al., 2000 [75, 76]	Baseline/follow-up
Energy Insecurity	Derived from Children’s HealthWatch Survey	Cook, et al. 2008 [1]	Baseline/follow-up
Housing Insecurity	Derived from Children’s HealthWatch Survey	Cutts, et al. 2011 [77]	Baseline/follow-up
Financial Behavior			
Financial capability^a^	Financial Capability Scale, Developed at University of Wisconsin Center for Financial Security	Collins, et al. 2013 [112]	Baseline/follow-up
Financial Behavior, Knowledge, and Self-efficacy^a^	4-section, 28-item survey	Danes, et al. 1999 [113]	Baseline/follow-up
Entrepreneurship and Savvy^a^	16-item survey question developed by Network study group	N/A	Baseline/follow-up
Physical and mental health related questions			
Adult’s Health			
Caregiver’s General Health	Self-rated physical health, self-rated physical health compared to the previous 3 months	N/A	Baseline/follow-up
Depression	10-item short version of CES-D scale	Radloff, et al., 1977 [80, 81, 82]	Baseline/follow-up
Substance Use	Alcohol abuse questions derived from Audit-C, drug abuse questions derived from DAST-10 screening test	Babor, et al. 2002 [114], Skinner, et al. [115]	Baseline/follow-up
Youngest Child’s Health			
Caregiver report of child’s health history	Derived from NHANES III survey	National Center for Health Statistics, 1990 [97]	Baseline/follow-up
Child Development	Parents’ Evaluation of Developmental Status survey	Glascoe, 1998 [84]	Baseline/follow-up
Exposure to Adversity and Violence			
Interaction with criminal justice	8-item survey	Brooks-Gunn, et al. 2011 [116, 81]	Baseline only
Exposure to community violence	Survey of Exposure to Community Violence 14-item survey	Richters, et al. 1990 [90]	Baseline only
Childhood adversity	Adverse Childhood Experiences 10-item survey	Felitti, et al. 1998 [89]	Baseline only
Social connection			
Social Support^a^	Social Support Network Scale 12-item survey	Block, et al. 2000 [117]	Baseline/follow-up
Social Capital^a^	Modified from Internet Social Capital Scales 20-item survey question	Williams, D. 2006 [118]	Baseline/follow-up
Program Evaluation			
Network Satisfaction	7-item survey developed by Network study group	N/A	Follow-up only

^a^Not included in this report on baseline outcomes

#### Career readiness

The career readiness outcomes included two measures: employment hope [72, 73], and general self-efficacy [74]. The employment hope scale was developed and validated by Hong et al., and consists of four components: psychological empowerment, future-oriented self-motivation, utilization of skills and resources, and goal-orientation [72, 73]. The general self-efficacy (GSE) scale was developed by Schwarzer and Jerusalem and found to be reliable and valid [74]. The GSE scale is widely used to assess individual self-efficacy in addressing daily challenges and adapting after stressful events.

#### Family economic hardship and physical and mental health

Family economic hardship is captured in three measures: the U.S Household Food Security Survey Module (HFSSM), an energy security survey, and a housing security survey. The HFSSM is a widely used, validated scale developed by U.S. Department of Agriculture to measure food insecurity, meaning the lack of access to enough food for an active and healthy life for the household and/or children [75, 76]. Eighteen questions are asked that include worry about not having enough money for food and reduction in diet quality over the course of 30 days. Household food insecurity consists of two dimensions: low food security, signified by food access problems and reduced quality of diet, and very low food security, signified by reduced food intake and disruption of eating patterns. The household energy security scale was developed and validated to measure access to adequate household heating and cooling [1]. The scale includes four questions to determine level of energy security in the household. Participants answered whether, in the past 3 months, the gas/electric company sent a letter threatening to shut off service for not paying bills, whether energy service was not delivered for not paying bills, whether there were any days in the past 3 months that the home was not heated/cooled because the household could not pay the bills, and whether the cooking stove was used to heat the home because the family could not pay bills. An affirmative response to only the first question (gas/electric company sent a letter threatening to shut-off service) indicates that a household has experienced “moderate energy insecurity”. For an affirmative answer to that question and at least one more, the household is considered to be severely energy insecure [1]. The housing security scale was developed and validated to assess access to adequate and stable housing, where housing insecurity is indicated by affirmative response to at least two of the following: overcrowding (more than 2 people per bedroom) or multiple moves (two or more moves in the previous year) [77, 78]. Overall, these three measures of hardship are related to poor self-rated health and depressive symptoms and poor child health and development [1, 77, 79].

We assessed participants’ self-rated mental and physical health, as well as caregiver-rated general health of the participants’ youngest child. Adult and child general physical health were identified by self-report health condition in four categories, excellent, good, fair, poor. To assess depressive symptoms among adult participants, we used the short version (10-items) depression screening scale developed by Center for Epidemiologic Studies Depression (CES-D) [80], which has been shown to be reliable and consistent with the original version [81, 82].

Child’s developmental risk were measured by the Parents’ Evaluation of Developmental Status survey [83], which has a sensitivity of 91–97 % and specificity of 73–86 % and has been validated with disadvantaged populations in the United States [84]. Participants were asked ten questions about their child’s developmental issues: global/cognitive, expressive language and articulation, fine-motor, gross motor, behavior, social-emotional, self-help, school, and any other concerns. Prior studies have suggested that two or more childhood developmental concerns can lead to major disability in adult life [85–87]. Glascoe’s study indicated that one in ten parents had at least two significant concerns about their child’s development [86]. These children for whom two significant concerns are reported at a young age are 20 times more likely to have a disability than children of parents who do not have any concerns [85].

#### Exposure to adversity and violence and criminal justice system

Exposure to childhood adversity was measured using the Adverse Childhood Experiences (ACEs) survey, a retrospective 10-item questionnaire that refers to experiences in the first 18 years of life [88, 89]. The ACEs survey is a widely used, validated survey to assess exposure to physical, emotional, and sexual abuse; physical and emotional neglect; and household dysfunction, including parental separation, domestic violence, and growing up with a household member who was mentally ill, abused substances, or was incarcerated. We assessed participants’ exposure to community violence with the Survey of Exposure to Community Violence (SECV), a validated scale developed by Richters et al. [90, 91] that includes reports of being a victim of or witness to community violence. We also measured participants’ interaction with the criminal justice system [92, 93].

### Statistical methods

We used SAS version 9.3 for all data analysis. All statistical tests are two sided. The baseline characteristics of participants in each study arm were assessed using Chi-square for categorical variables and simple t-tests for numerical variables.

## Results

### Demographics

Of the 103 participants that participated in the baseline survey, 37 were in the full intervention group, 35 in the partial intervention group, and 31 in the control group. Table 2 presents basic characteristics of all participants. The mean age for participants was 25 years old. The youngest child’s mean age was 30 months. Majority of participants were women (94.2 %), US-born (98.1 %), Black non-Hispanic (88.4 %), never married (83.5 %), unemployed (94.2 %), and without a bank account (66 %). Our baseline data indicates that there were no statistically significant differences in social or economic conditions among participants in the three study arms in demographics or other measures.

**Table 2:** Baseline Characteristics of the Participants of Building Wealth and Health Network Randomized Controlled Trial

			Group Assignment						
	Total		Control Group		Partial Intervention		Full Intervention		Significance
	*N* = 103		*N* = 31		*N* = 35		*N* = 37		
	Mean	SD	Mean	SD	Mean	SD	Mean	SD	*p*-value
Child’s age (months) Missing = 8	30.4	18.7	30.9	16.0	29.1	17.8	31.3	21.5	0.80^a^
Caregiver’s age (years)	25.4	5.2	26.4	4.3	24.6	5.6	25.3	5.5	0.07^a^
	*N*	%	*N*	%	*N*	%	*N*	%	*N*
Caregiver’s gender									0.65^b^
Male	6	5.83	1	3.2	3	8.6	2	5.4	
Female	97	94.2	30	96.8	32	91.4	35	94.6	
Immigration status									0.64^b^
US born	101	98.1	36	97.3	34	97.1	31	100.0	
Foreign born	2	1.94	1	2.7	1	2.9	0		
Race/Ethnicity									0.11^b^
Hispanic	5	4.9	3	9.7	1	2.9	1	2.7	
Black non Hispanic	91	88.4	25	80.7	30	85.7	36	97.3	
White non Hispanic	2	1.9	0		2	5.7	0		
Other	5	4.9	3	9.7	2	5.7	0		
Sexual orientation									0.52^b^
Heterosexual or straight	86	83.5	24	77.4	29	82.9	33	89.2	
Gay or lesbian	3	2.9	1	3.2	2	5.7	0		
Bisexual	14	13.6	6	19.4	4	11.4	4	10.8	
Marital status									0.73^b^
Married	1	1.0	0		0		1	2.7	
Separated	4	3.9	0		2	5.7	2	5.4	
Never married	86	83.5	27	87.1	28	80.0	31	83.8	
Living with partner	12	11.7	4	12.9	5	14.3	3	8.1	
Education									0.82^b^
Some high school or grade school	30	29.1	7	22.6	11	31.4	12	32.4	
High school graduate or GED	35	34.0	11	35.5	14	40.0	10	27.0	
Technical school or some college and above	38	36.9	13	41.9	10	28.6	15	40.5	
Employment									0.47^b^
Unemployed	97	94.2	36	97.3	33	94.3	28	0.3	
Employed	6	5.8	1	2.7	2	5.7	3	9.7	
Banking									0.43▲
Have an open bank account	35	34.0	13	41.9	12	34.3	10	27.0	
Do not have an open bank account	68	66.0	18	58.1	23	65.7	27	73.0	

^a^tested by Wilcoxon-Mann–Whitney test

^b^tested by Fisher’s Exact test

▲tested by Chi-square test

### Career readiness

Career readiness characteristics are outlined in Table 3. Network participant reports of employment hope are clustered at the top end of the scale with a mean of 128.3 out of 140 maximum score, with over 20 % reaching the maximum score. The employment hope scale is a new scale and there are no nationally representative norms available yet for comparison. The mean for General Self-Efficacy reported at baseline was 31.1 out of a maximum 40 on the scale. This is similar and within range of the mean of 29.5 that has been reported for the general population of the United States [94].

**Table 3:** Career Readiness

Employment Hope	Mean	SD
When working or looking for a job, I am respectful towards who I am.	9.7	1.0
I am worthy of working in a good job.	9.7	1.0
I am capable of working in a good job.	9.8	0.7
I have the strength to overcome any obstacles when it comes to working.	9.4	1.2
I am going to be working in a career job.	9.2	1.7
I feel energized when I think about future achievement with my job.	9.3	1.5
I am aware of what my skills are to be employed in a good job.	9.5	1.3
I am aware of what my resources are to be employed in a good job.	9.0	1.7
I am able to utilize my skills to move toward career goals.	9.2	1.6
I am able to utilize my resources to move toward career goals.	9.0	1.7
I am on the road toward my career goals.	8	2.7
I am in the process of moving forward reaching my goals.	8.7	2.0
Even if I am not able to achieve my financial goals right away, I will find a way to get there.	9.4	1.3
My current path will take me to where I need to be in my career.	8.5	2.3
General Self-Efficacy	31.1	4.9
I can always manage to solve difficult problems if I try hard enough.	3.5	0.6
If someone is against me, I can find ways to get what I want.	2.5	1.0
It is easy for me to stick to my aims and reach my goals.	3.2	0.8
I am confident that I could deal with unexpected events.	3.1	0.7
Thanks to my resourcefulness, I know how to handle unforeseen situations that I don’t expect.	3.1	0.8
I can solve most problems if I put in the necessary effort.	3.4	0.7
I can remain calm when facing difficulties because I can rely on my coping abilities.	3.0	0.9
When I am faced with a problem, I can usually find several solutions.	3.0	0.8
If I am in trouble, I can usually think of a solution.	3.1	0.8
I can usually handle whatever comes my way.	3.2	0.7

### Family economic hardship and health and wellbeing

At baseline, participants reported economic hardship at higher rates than the general population. As seen in Table 4, over 30 % of participants reported very low household food security, over five times the national rate of 5.9 % for households with children [76]. About 65 % of participants at baseline reported housing insecurity which includes crowding and/or multiple moves. This is significantly higher than that reported by Cutts et al.’s study in 2011, which indicated that 46 % of families with children under age four, reporting to clinical settings, experienced housing insecurity [77]. A significant percentage (31.1 %) of participants also reported severe energy insecurity, which is associated with mental distress and depression [95]. This prevalence rate for severe energy insecurity is much higher than the prevalence of severe energy insecurity compared to the 23 % observed in Cook et al.’s 2008 study with caregivers of young children [96].

**Table 4:** Hardship and Health

Hardship	Number	Percent
Food security status		
Food secure	45	43.7
Low food secure	25	24.3
Very low food secure	33	32.0
Housing security		
Housing secure	36	35.0
Housing Insecure	67	65.0
Energy security		
Energy or moderate energy secure	71	68.9
Severe energy insecure	32	31.1
Physical and Mental Health		
Caregiver’s Health	Number	Percent
Adult self-rated health		
Excellent or good	68	66.0
Fair or poor	35	34.0
Adult self-report depression (CES-D)		
Depression	59	57.3
No depression	44	42.7
Child’s Health		
Adult-rated child’s health		
Excellent or good	81	78.6
Fair or poor	22	21.3
Parents' Evaluation of Developmental Status		
No significant developmental concern	71	70.3
One significant developmental concern	16	15.8
More than one significant developmental concern	11	10.9

Slightly over a third (34 %) of adult participants rated their health as fair or poor. This prevalence of fair/poor health is twice that of the general US population [97]. The prevalence of depression (57.3 %) is more than twice that found in the nationally representative NHANES study [98], and more than eight times the rate reported in the Diagnostic and Statistical Manual of Mental Disorders (DSM) [99]. Results for the health and wellbeing of the participants’ youngest child (under age 6), show that over 20 % of the participants considered their youngest child’s health as fair or poor, which is significantly higher than the national prevalence rates for children aged 5 years and younger [100]. Additionally, 26 % reported their youngest child had at least one developmental concern, while 10 % reported two or more concerns, for a pooled prevalence rate of 36 %, which is higher than pooled prevalence estimate of 31.5 % (95 % CI 27.0–36.0 %) from a meta-analysis of 37 population-based studies [101].

### Exposure to adversity and violence and interaction with criminal justice

As seen in Table 5, over 35 % of the participants had four or more ACEs, compared to 6.2 % in a study of 9508 patients in the Kaiser Permanente health care system [102]. The most prevalent experiences included parental separation (71 %), substance abuse of a household member (43 %), emotional abuse (37 %), and sexual abuse (18 %). These individually reported rates are higher than individual reports recently reported in a population-based sample of Philadelphia residents, where 34.8 % reported substance abuse by household members, 33.2 % reported emotional abuse, and 16.2 % reported sexual abuse [103].

**Table 5:** Exposure to Adversity and Trauma & Criminal Justice

Adult’s adverse childhood experiences (ACEs)	Number	Percent
0 ACE	15	14.6
1-3 ACEs	48	46.6
≥ 4 ACEs	40	38.8
Individual adverse childhood experience		
Emotional Abuse	38	36.9
Physical Abuse	28	27.2
Sexual Abuse	18	17.5
Emotional Neglect	37	35.9
Physical Neglect	18	17.5
Parents Separated/Divorced	73	70.9
Mother Abused	22	21.4
Household Substance Abuse	44	42.7
Household Mental Illness	20	19.4
Household Incarceration	32	31.1
Survey of Exposure to Community Violence		
Exposure to Violence	Number	Percent
Ever been picked-up, arrested, or taken away by the police	45	43.7
Ever been threatened with serious physical harm by someone	43	41.7
Ever been slapped, punched, or hit by someone	62	60.2
Ever been beaten up or mugged	31	30.1
Ever been attacked or stabbed with a knife	18	17.5
Ever been shot with a gun	7	6.8
Witnessing of violence		
Ever saw someone else getting beaten	61	60.4
Ever heard the sound of gunfire near home	88	86.3
Ever saw a seriously wounded person after an incident of violence	66	64.7
Ever saw someone else being attacked or stabbed with a knife	38	36.9
Ever saw someone else get shot with a gun	52	51.0
Ever saw a dead person somewhere in the community (besides wakes and funerals)	49	48.0
Ever saw someone being killed by another person	28	27.2
Ever heard about someone being killed by another person	82	80.4
Interaction with Criminal Justice System	Number	Percent
Did you ever spend time in a youth correctional institution like a training school or reform school?	15	14.6
	Mean	SD
*If “yes” to above: Altogether, how much time did you spend there?*	2.2	1.0
	Number	Percent
Did you ever spend time in an adult correctional institution like a county, state or federal jail or prison?	15	14.6
	Mean	SD
*If “yes” to above: Altogether, how much time did you serve in this adult institution?*	1.3	0.05
	Number	Percent
Did the father of your child spend any time in jail or prison?	50	48.5
Did the mother of your child spend any time in jail or prison?	1	1.0

As measured on the Survey of Exposure to Community Violence (SECV), the majority of participants had experienced serious community violence. For example, 60 % of participants reported being slapped, punched, or hit by someone, 30 % reported they had been beaten up or mugged and over 17 % reported being attacked or stabbed with a knife. Additionally, witnessing violence was also prevalent, as 86 % heard sound of gunfire near their homes, 65 % saw a seriously wounded person after an incident of violence, and 27 % had seen someone killed. These findings are comparable to other research on violence exposure among very low-income parents. For example, one study also using the SECV among adults found that 25 % of the sample had been beaten up or mugged, and a third had seen someone killed [104]. Using a similar measure, the Fragile Families study showed moderate levels of community violence exposure in 29 % of respondents, and high levels in 20 % [105].

Our baseline findings show that 15 participants (14.6 %) have spent time in an adult correctional institution, with a mean duration of over 2 years and nearly half (48.5 %) of the fathers of the participants’ youngest child has spent time in prison, well above the state average. According to the U.S. Department of Justice, 2.4 % of adult females and 3.3 % of adult males in Pennsylvania were incarcerated in 2013 [106].

## Discussion

The Network RCT is designed to measure the effectiveness of an intervention consisting of financial empowerment education in isolation and in combination with mental health support among TANF recipients with young children. Our baseline data indicates that there were no statistically significant differences in social or economic conditions among the three study arms, which suggested all participants at baseline have been successfully randomized into three study groups.

Variables related to career readiness, employment hope and general self-efficacy do show some promise for the potential of these TANF participants, and may serve as an indication of their potential to succeed in the Building Wealth and Health Network intervention program. Taken together, however, the other baseline data from Network participants paints a bleak picture of severe childhood adversity, adult violence exposure, and multiple poverty-related stressors. Very high levels of food insecurity, housing insecurity, and energy insecurity reflect profound economic hardship among this group of young parents receiving TANF. These too are related to depression, which is reflected in the high rate of depression, where over half of the participants meet the clinical criteria. The high rates of poor health and development of the children in these families reflect the adversity to which they are exposed. Overall, the levels of hardship were significantly higher than that reported in the general population, and in most instances comparable to other very low-income populations.

Despite all of these hardships, every participant in the Network RCT was deemed “work mandatory” and was being held accountable to the 20 h per week work participation requirement. Given the level of hardship and adversity, and the high-risk nature of their housing and nutrition situations, including exposure to violence, there should be improved screening procedures to deem a person work-ready/work mandatory. Additionally, front-line caseworkers should be empowered to provide a more coordinated, wrap around approach to providing services for at-risk families, and greater support that goes beyond assigning job search activities.

In spite of multiple stressors and adversities, Network RCT participants maintain fairly high levels of self-efficacy and employment hope. These strengths can be drawn upon with appropriate programming to support the aspirations and goals of participants. The Building Wealth and Health Network attempts to incorporate these strengths through building resilience, social support, and opportunities to express self-efficacy.

Due to the nature of our intervention program, we were not able to achieve double blinding in the randomized controlled trial. However, all staff and instructors were trained to minimize bias and to keep all information from other intervention arms confidential during the study. Our study is limited by a small sample size at baseline. However, implementation of missing value imputation might improve power in the later analysis.

There are several strengths in our study. Our rigorous recruitment and research methods demonstrate that a small-scale RCT in a highly complex environment with participants who have major hardships and barriers to employment can be effective. The intervention itself, which combines matched savings, financial empowerment education, and trauma-informed peer support, is a major innovation to TANF. Our baseline data provides insightful information regarding financial hardship, physical and mental wellbeing and exposure to violence that is rarely considered together for TANF recipients.

## Conclusion

These families with young children participating in TANF demonstrate very high levels of adversity and economic hardship, suggesting that safety net programs meant to encourage participation in the workforce should utilize comprehensive and robust approaches to help families access treatment for depression and overcome serious economic hardship related to poor housing and nutrition. Given the high levels of adversity and violence exposure, adding trauma-informed approaches to social services and job search assistance may show demonstrable promise [107, 108]. Two-generation interventions that address the mental health and economic stability of caregivers simultaneously, with the health and development of children, may offer opportunities for successful and stable entry into the workforce [109, 110].

## Abbreviations

ACASI, Audio Computer-Assisted Self-Interview; ACE, Adverse Childhood Experiences; CAO, County Assistant Office; CWDS, Commonwealth Workforce Development System; GSE, General self-efficacy; HFSSM, U.S Household Food Security Survey Module; RCT, Randomized controlled trial; SECV, Survey of Exposure to Community Violence; SEG, Self Empowerment Group; SNAP, Supplemental Nutrition Assistance Program; TANF, Temporary Assistance for Needy Families; WIC, Special Supplemental Nutrition Program for Women, Infants, and Children
